# Supplementary material for: Inducing skyrmions in ultrathin Fe films by hydrogen exposure
Source: Nat Commun. 2018 Apr 20;9:1571. doi: 10.1038/s41467-018-04015-z (PMC5910423; doi:10.1038/s41467-018-04015-z)
Supplement: Supplementary file 1 — Supplementary Information [file 41467_2018_4015_MOESM1_ESM.pdf]

Supplementary Information

Hsu, P.-J. et al. Inducing skyrmions in ultrathin Fe films by hydrogen exposure.

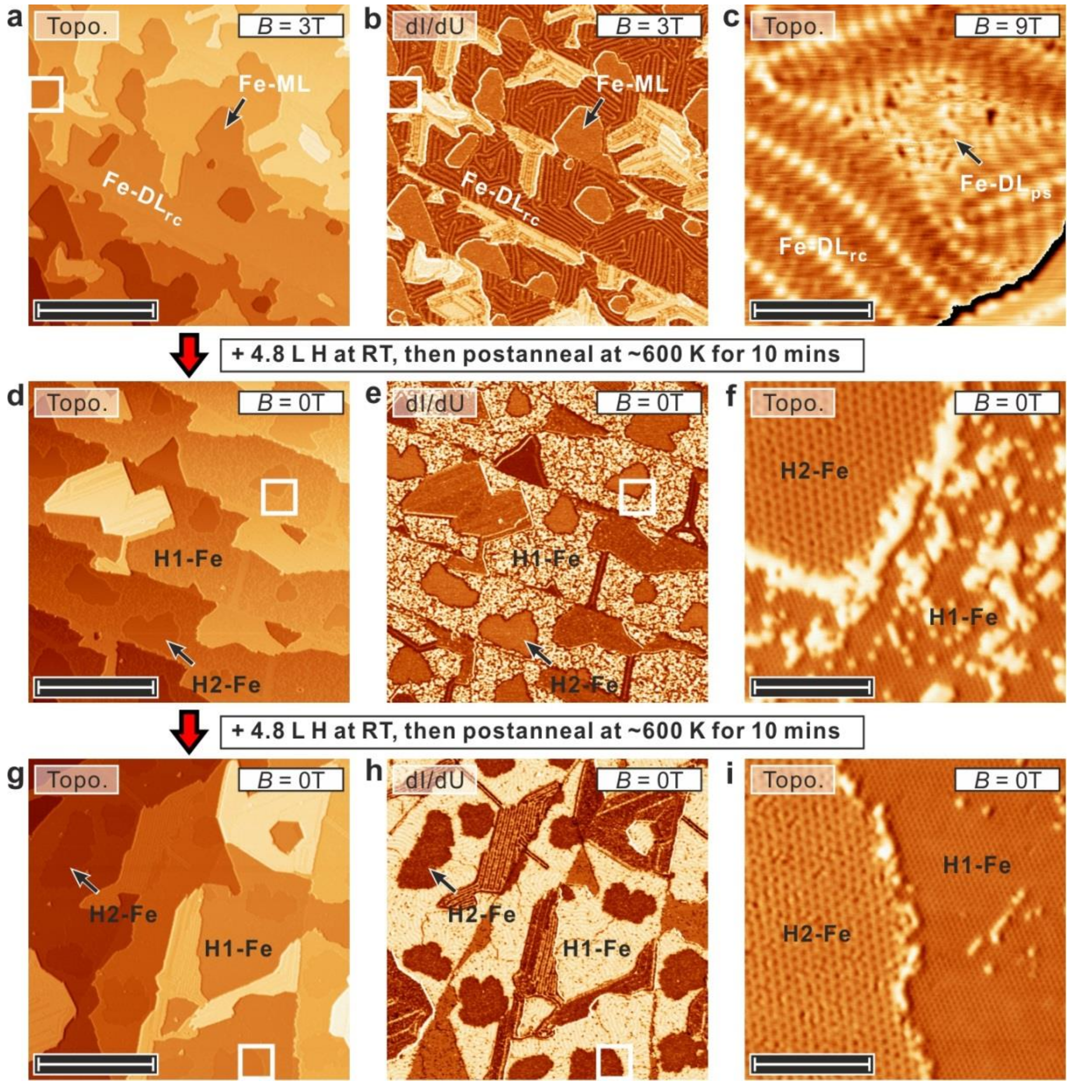

Supplementary Figure 1 | Hydrogenation of the Fe double layer on Ir(111). **a**,-**c**, SP-STM constant-current image, dI/dU map, and magnified view of about 2.2 atomic layers of Fe on Ir(111). The Fe was deposited with the Ir substrate held at about 500 K. **d**,-**f**, Same as in panels **a**-**c** after exposure of the same sample to 4.8 L of atomic hydrogen (4 min at pressure of $2 \cdot 10^{-8}$ mbar) at room temperature (the sample was removed from the 4.2 K STM about 1 h before exposure). After the hydrogen exposure the sample was annealed at about 600 K for 10 min. **g**,-**i**, Same as in panels **d**-**f** after repeating the same procedure as before (i.e. 1 h to reach room temperature, exposure to 4.8 L hydrogen, post-annealing at about 600 K). Measurement parameters: U = +1.0 V for panels **a**,**b**,**d**,**e**,**g**,**h** and U = -0.2 V for panels **c**,**f**,**i**; I = 1 nA; T = 4-5 K; Cr bulk tip; scale bars 100 nm for panels **a**,**b**,**d**,**e**,**g**,**h**; scale bars 10 nm for panels **c**,**f**,**i**.

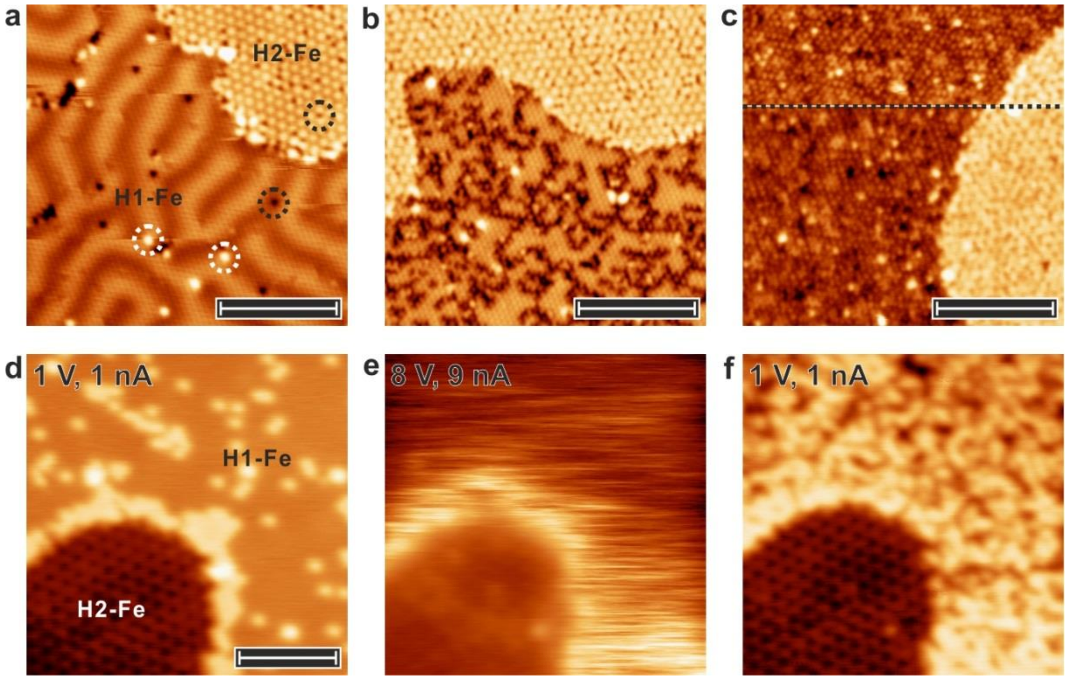

Supplementary Figure 2 | Disordered H-Fe phases and tip-induced disorder. a-c, Constant-current images of a measurement series with repeated exposure of Fe/Ir(111) to H at room temperature and subsequent annealing at about 600 K for 10 min: **a**, after exposure to 4.8 L of H; bright protrusions (hydrogen vacancies) and dark pits (extra hydrogen atoms) are highlighted by white and black dashed circles, respectively. **b**, after additional 4.8 L of H; **c**, after additional 48 L of H. The black dashed line indicates a tip change during scanning. Regardless of the degree of disorder within the superstructures, the boundary between the H1-Fe and H2-Fe areas remains abrupt. **d-f**, Constant-current images of a measurement series with changing tunnel parameters as indicated in the panels; comparison between panels **d** and **f** demonstrates a change of the H1-Fe phase due to the extreme tunneling conditions in panel **e**. Measurement parameters for panels **a-c**: U = -0.5 V; I = 1 nA; all: T = 4-5 K; Cr bulk tip; scale bars 10 nm for panels **a-c**; scale bar 6 nm for panel **d**.

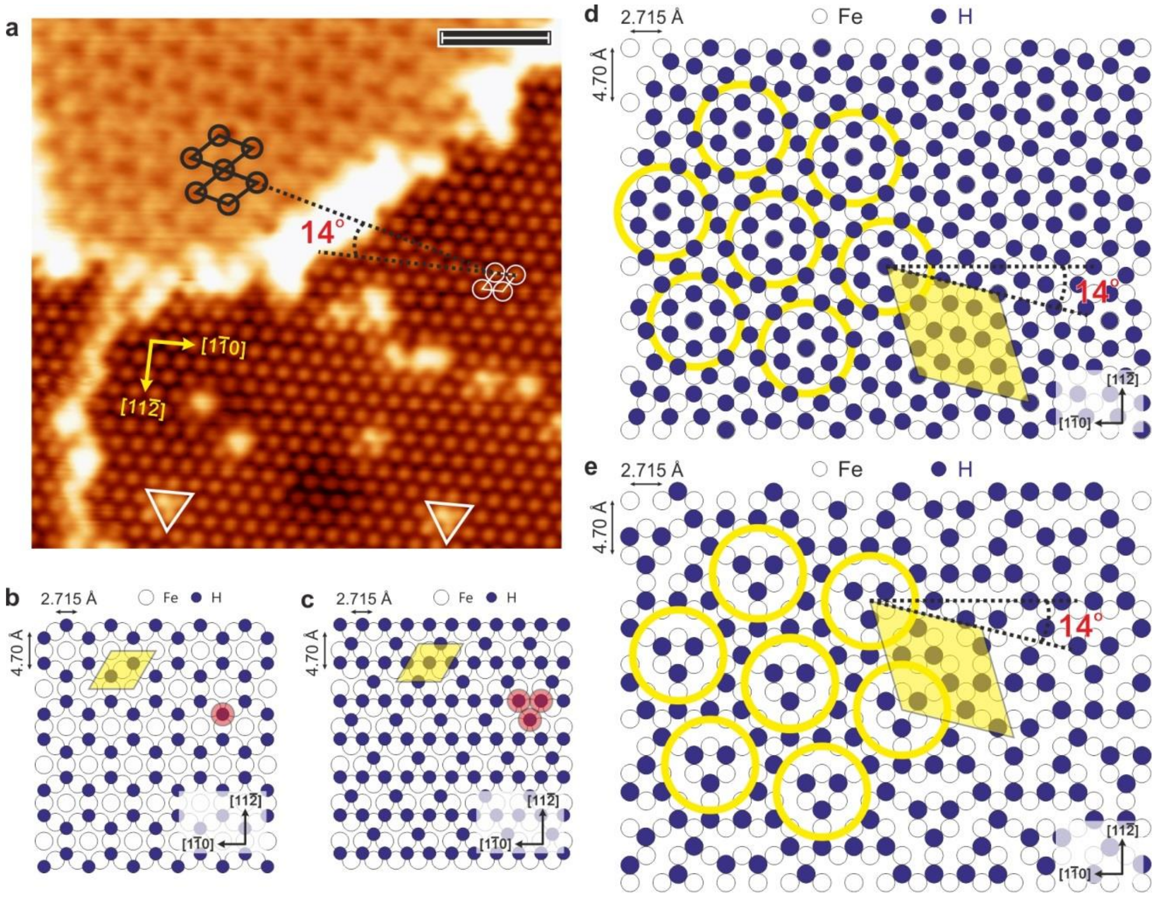

Supplementary Figure 3 | Possible structural models of the H-induced superstructures displayed for a single hexagonal Fe layer. a, High resolution STM constant-current topography image of H1-Fe and H2-Fe. The angle between the close-packed directions of the hexagonal lattices observable for the superstructures is 14°. The hydrogen vacancies (bright protrusions) with the same geometrical shape have been marked by white triangles. Scale bar 3 nm. **b**,**c**, Possible H1-Fe superstructure models for 0.50 ML and 0.75 ML H coverages, respectively. Yellow diamonds denote the unit cell of the superstructure, which is aligned along the high-symmetry lines of the Ir (111) surface due to the $p(2 \times 2)$ structure. The solid red circles indicate possible missing H atoms for the hydrogen vacancies shown in panel **a**. **d**,**e**, Possible H2-Fe superstructure models with 16 and 9 H atoms in a unit cell of 13 Fe atoms. Yellow diamonds denote the unit cells of the H2-Fe structure, while yellow circles illustrate the hexagonal lattice of the superstructure.

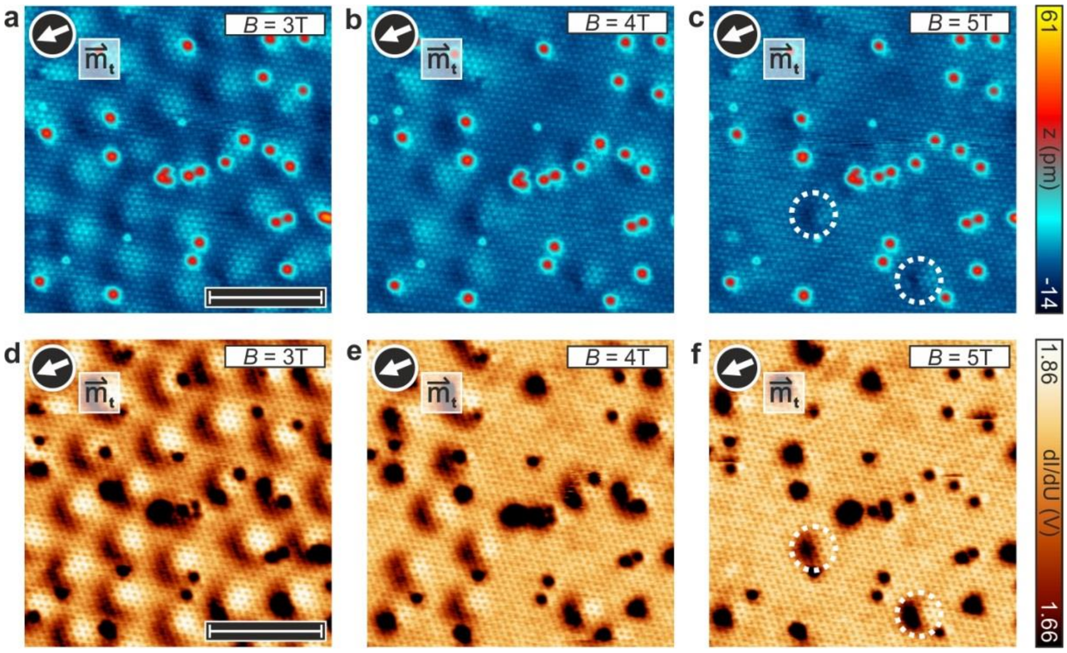

Supplementary Figure 4 | Identification of skyrmions and defects. a,-c, Spin-polarized constant-current images of the same sample area at different applied magnetic fields as indicated in the panels. Defects appear as protrusions and magnetic skyrmions can be identified by their two-lobe structure (brighter to the top right and darker to the bottom left). The number of skyrmions decreases as the magnetic field is increased, see white dashed circles in panel **c** for some skyrmions remaining at 5 T. **d,-f**, Maps of differential tunneling conductance acquired simultaneously with the constant-current images; at this bias voltage the defects appear dark, whereas the magnetic skyrmions have a very similar appearance compared to the constant-current images. Measurement parameters: U = -0.2 V; I = 1.0 nA; T = 4.2 K; Cr bulk tip; scale bars 8 nm.

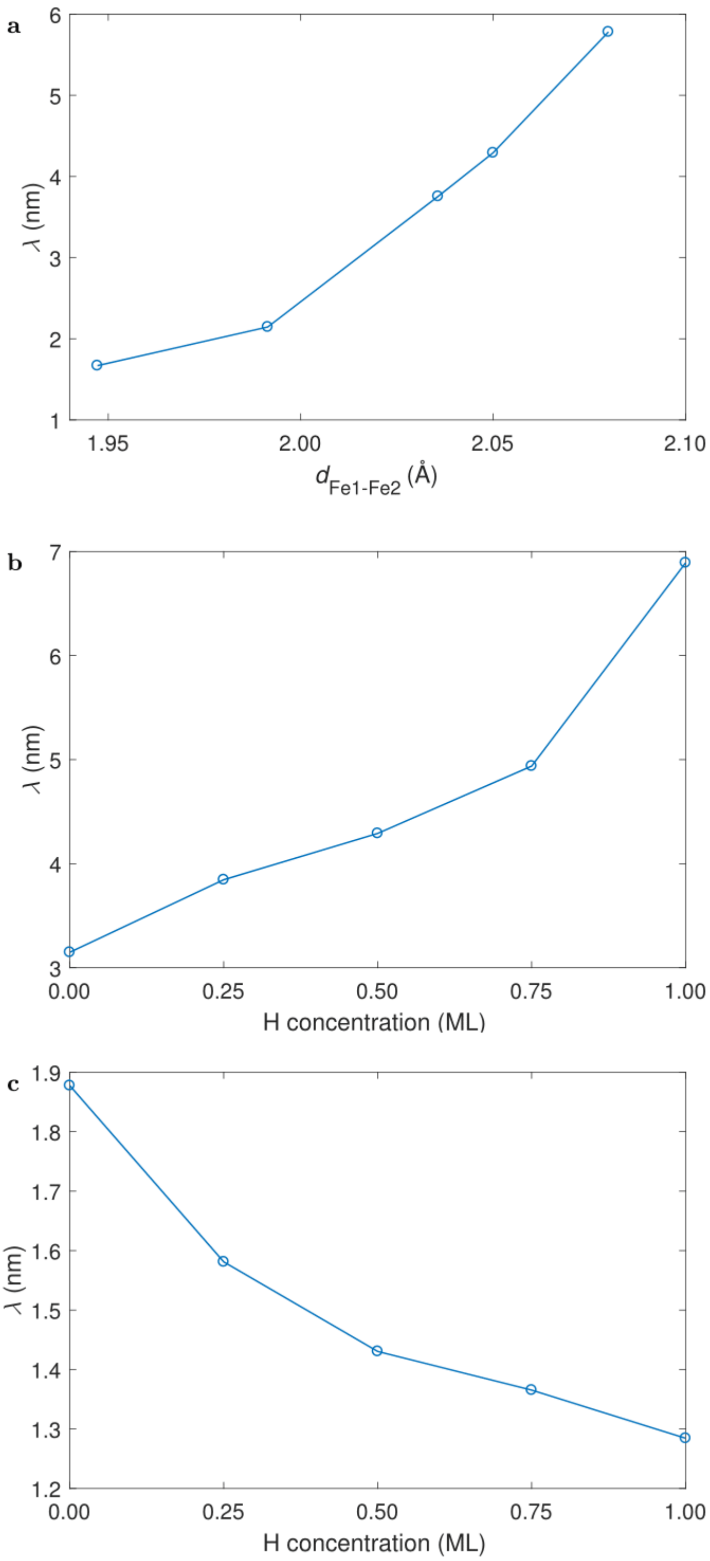

Supplementary Figure 5 | Dependence of the spin spiral period on calculation parameters. a, Dependence of the period on the Fe1-Fe2 interlayer distance, with the other layer distances and H concentration kept fixed at the value for the system modelling the H1-Fe structure (0.50 ML H coverage in Fe1-Fe2 A position). **b**, Dependence of the spin spiral period on the H concentration, with the other parameters again kept fixed at the values for the H1-Fe model. **c,** Dependence of the spin spiral period on the H concentration, with the fixed geometry of the H in the Fe2-Vac A position.

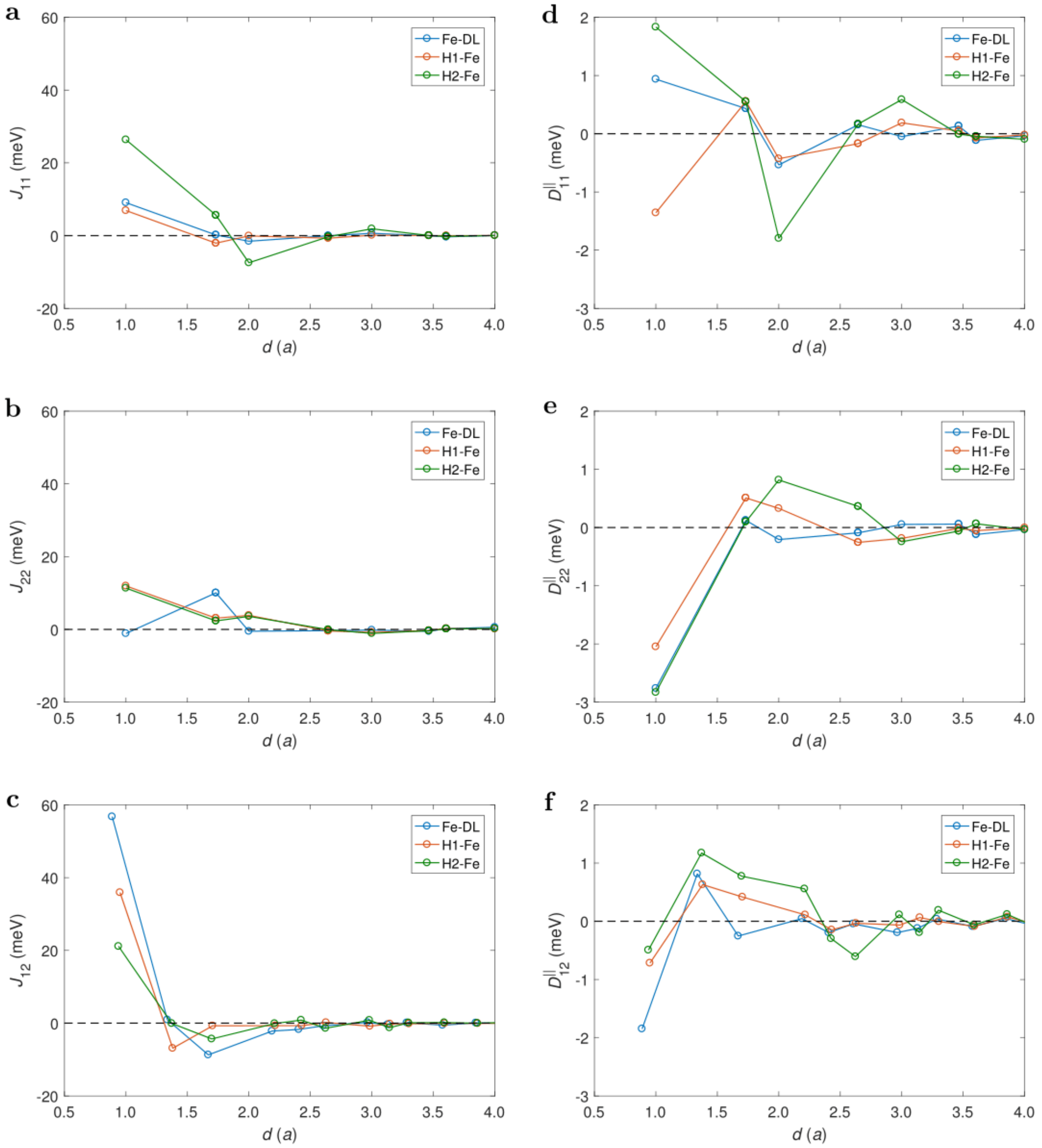

Supplementary Figure 6 | Spin interaction parameters calculated from the relativistic torque method with the SKKR program. a,-c, Heisenberg exchange interactions within **a**, the first and **b**, the second Fe layers, as well as **c**, between the two Fe layers as a function of distance between the neighbours. The values are given for the Fe-DL, H1-Fe (0.50 ML H at Fe1-Fe2 A) and H2-Fe (H at Ir-Fe1 C) systems investigated in the main text. Positive and negative values denote ferromagnetic and antiferromagnetic couplings, respectively. **d,-f**, In-plane component of the Dzyaloshinsky–Moriya vectors. Positive and negative values prefer left- and right-handed rotations, respectively.

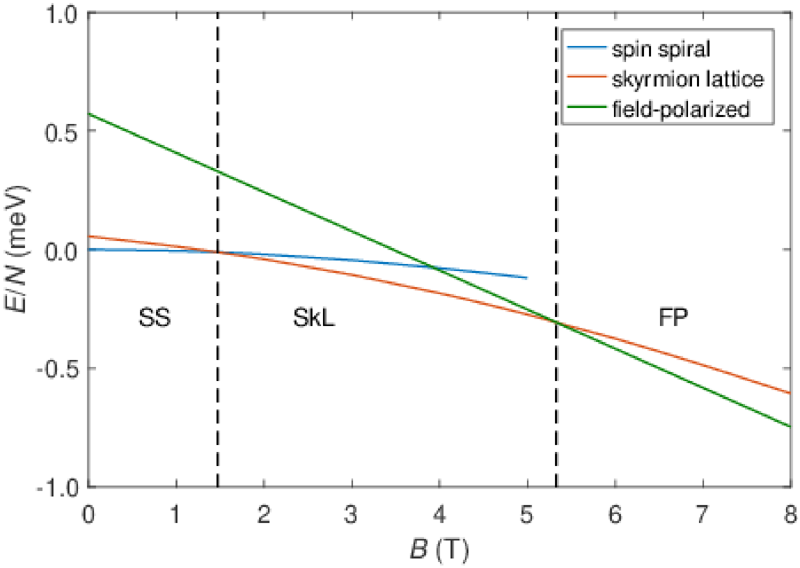

Supplementary Figure 7 | Zero-temperature phase diagram calculated from the *ab initio* interaction parameters. The interaction parameters are displayed in Supplementary Fig. 6 for the H1-Fe system. The phase diagram shows reasonable quantitative agreement with the one determined using the simplified model parameters, Fig. 5 in the main text.

Ir/Fe1/Fe2	E (eV)	Fe1 μ (μ_B)	Fe2 μ (μ_B)	Ir-Fe1 d (Å)	Fe1-Fe2 d (Å)
A/B/C	0.000	2.45	2.70	2.09	1.82
A/B/A	0.070	2.31	2.67	2.10	1.78
A/C/B	0.017	2.50	2.71	2.09	1.84
A/C/A	0.096	2.35	2.67	2.10	1.80

Supplementary Table 1 | Results of VASP calculations for different stackings of the Fe double layer. The table displays energies E, magnetic moments μ and interlayer distances d. The energies are given with respect to the lowest energy A/B/C stacking.

H position	E (eV)	Fe1 μ (μ_B)	Fe2 μ (μ_B)	Ir-Fe1 d (Å)	Fe1-Fe2 d (Å)	H Q (e)
Fe2-Vac A	0.000	2.45	2.27	2.09	1.90	1.22
Fe2-Vac B	0.033	2.50	2.27	2.09	1.89	1.22
Fe2-Vac C	1.127	2.42	2.36	2.06	2.01	0.96
Fe1-Fe2 A	0.616	2.33	2.38	2.08	2.11	1.36
Fe1-Fe2 B	0.671	2.49	2.16	2.06	2.10	1.36
Fe1-Fe2 C	0.848	2.15	2.79	2.12	2.15	1.38
Ir-Fe1 A	1.055	2.29	2.76	2.50	1.85	1.24
Ir-Fe1 B	1.652	2.52	2.65	2.68	1.74	0.92
Ir-Fe1 C	1.158	2.07	2.83	2.27	2.01	1.21

Supplementary Table 2 | Results of VASP calculations for different H adsorption sites on the Fe double layer. The stacking for the Fe-DL was A/B/C. The table displays energies E, magnetic moments μ, interlayer distances d and Bader charges Q. The energies are given with respect to the lowest energy Fe2-Vac A adsorption site.

H coverage	Fe1 μ (μ_B)	Fe2 μ (μ_B)	Ir-Fe1 d (Å)	Fe1-Fe2 d (Å)	H Q (e)
0.00 ML	2.45	2.70	2.09	1.82	0.00
0.25 ML	2.45	2.69	2.08	1.96	1.39
0.50 ML	2.40	2.62	2.07	2.05	1.38
0.75 ML	2.36	2.50	2.07	2.09	1.37
1.00 ML	2.33	2.38	2.08	2.11	1.36

Supplementary Table 3 | Results of VASP calculations for different H concentrations on the Fe double layer. The Fe1-Fe2 A (octahedral between the layers) adsorption site was considered for H. The 0.00 ML coverage corresponds to the A/B/C stacking of the Fe layers in the pristine Fe-DL from Supplementary Table 1. The 1.00 ML coverage corresponds to the Fe1-Fe2 A position from Supplementary Table 2. The table displays magnetic moments μ, interlayer distances d and Bader charges Q. The magnetic moments and the vertical positions were averaged over Ir, Fe and H atoms belonging to the same atomic layer in the p(2×2) unit cell.

H position	Fe1 μ_S (μ_B)	Fe2 μ_S (μ_B)	H Q (e)
Fe-DL	2.87	2.92	0.00
Fe2-Vac A	2.50	2.93	1.22
Fe1-Fe2 A	2.71	2.66	1.36
Ir-Fe1 C	2.37	2.77	1.19
H coverage	Fe1 μ_S (μ_B)	Fe2 μ_S (μ_B)	H Q (e)
0.00 ML	2.87	2.92	0.00
0.25 ML	2.90	2.96	1.44
0.50 ML	2.83	2.86	1.41
0.75 ML	2.76	2.76	1.38
1.00 ML	2.71	2.66	1.36

Supplementary Table 4 | Results of SKKR calculations for different H adsorption sites and concentrations. The table displays spin magnetic moments μ_S and charges Q, compare Supplementary Tables 2-3 for the VASP data. The 0.00 ML and 1.00 ML coverages correspond to the Fe-DL and Fe1-Fe2 A rows in the H position table, respectively.

system	$\mathcal{J}_1$ (10^{-12}J m^{-1})	$\mathcal{J}_2$ (10^{-30}J m)	$\mathcal{D}$ (10^{-3}J m^{-2})	$\mathcal{K}$ (10^{6}J m^{-3})
Fe-DL	-12.69	0.39	-6.99	3.16
H1-Fe	0.00	0.35	-6.05	-0.13
H2-Fe	4.35	0.37	-1.46	3.34

Supplementary Table 5 | Micromagnetic parameters for the model systems. Negative values of $\mathcal{J}_1$ indicate a spin spiral state formed by the frustrated Heisenberg exchange interactions, cf. $f = (J_1 + 3J_2)/J_1$ in Table 1 of the main text with the atomistic parameters. Negative sign of $\mathcal{D}$ prefers a right-handed rotation of the spins. Positive and negative signs of $\mathcal{K}$ denote easy-axis and easy-plane anisotropies, respectively.

Supplementary Note 1 | The different hydrogen-induced phases H1-Fe and H2-Fe.

The growth of Fe on Ir(111) changes with layer thickness. Whereas the first monolayer grows pseudomorphically with respect to the substrate[1], the second monolayer releases strain by the incorporation of dislocation lines along the $[11\overline{2}]$ directions[2]. Supplementary Figures 1a and 1b show a constant-current image and a simultaneously acquired dI/dU map, respectively, of a sample of about 2.2 atomic layers of Fe on Ir(111). On the Fe double layer (Fe-DL) the dislocation lines form arrays with distances of about 5 nm. Some pseudomorphic Fe-DL regions exist between these arrays. The magnified view presented in Supplementary Fig. 1c demonstrates that the magnetic spin spiral in the reconstructed Fe-DL is unchanged by external magnetic fields up to 9 T.

When atomic hydrogen is dosed onto this sample at room temperature (about 1 hour after removal from the 4.2 K STM) and the sample is annealed afterwards, the morphology changes, see Supplementary Figs. 1d-f: the number of dislocation lines is strongly reduced[3], most of the Fe-DL area exhibits a $p(2 \times 2)$ superstructure (H1-Fe) with several defects, and some islands with a 0.98 nm periodic superstructure (H2-Fe) are formed. A similar disappearance of reconstructed areas on the Ir(100) surface upon hydrogen adsorption has been demonstrated earlier[4], attributed to the saturation of the truncated bonds of surface atoms by the adsorbant. Upon additional exposure of the same sample to hydrogen at room temperature and subsequent annealing, the sample changes again, as seen in Supplementary Figs. 1g-i: the H1-Fe phase is now well-ordered, i.e. the amount of hydrogen is now sufficient to form the $p(2 \times 2)$ superstructure. The H2-Fe islands are now larger, but the ratio of the two areas has not changed. We conclude that the two phases cannot easily be transformed into each other. However, since the H2-Fe islands can coalesce during annealing, this also leads to well-defined and more extended areas of the H1-Fe structure where skyrmions can form under the application of an external field. Such a sample was used for the investigations shown in Fig. 2 of the main text.

Based on our measurements we can derive neither the H concentration nor the vertical or lateral adsorption sites of the H atoms. However, we find that there is always a sharp boundary between the two H-Fe superstructures, even if one or both of the phases are rather disordered due to insufficient or excessive H exposure or treatment at very high temperature, see Supplementary Figs. 2a-c. If the two phases would differ in their H concentration only, a more gradual transition between the two would be expected, in particular when a phase is already disordered. Thus we suspect that the two H-Fe superstructures originate from different vertical positions of the H atoms with respect to the surface.

The repeated dosages of hydrogen also shed light on the origin of two types of point defects, namely bright protrusions and dark pits in constant-current topography images. By comparing Supplementary Figs. 1f and 1i, it can be observed that the number of bright protrusions on top of the H1-Fe and at the edge of the H2-Fe drops significantly after exposure to an additional amount of 4.8 L hydrogen, which allows us to identify the nature of these protrusions as hydrogen vacancies. Once the H1-Fe and the H2-Fe have incorporated a sufficient amount of atomic hydrogen, further hydrogen exposure will turn them into disordered phases and the additional hydrogen atoms on top of them appear as dark pits as shown in Supplementary Figs. 2a and b. In Supplementary Fig. 2a, the hydrogen vacancies (bright protrusions) and extra hydrogen atoms (dark pits) have been indicated by white and black dashed circles, respectively.

The different impact of measurements at high bias voltage on the two phases also suggests different adsorption sites, see measurement series in Supplementary Figs. 2d-f: First, a sample area with both H1-Fe and H2-Fe is imaged with tunneling parameters of U = 1.0 V and I = 1 nA, see panel d. Next, the same area is scanned with a high voltage of U = 8.0 V and a high current of I = 9 nA, see panel e. Finally, with the same imaging parameters as used in panel d, it becomes evident that these extreme tunneling parameters strongly affected the H1-Fe phase, where a tip-induced disorder is observed, whereas the H2-Fe is hardly altered, compare panels d and f. This suggests that the hydrogen in the H2-Fe phase is bound much stronger, possibly closer to the Ir-Fe interface than in the H1-Fe phase.

The observed superstructures allow for several different structural models with different H concentrations, several of which are visualized in Supplementary Fig. 3. The p(2×2) H1-Fe could be realized by H atoms in both fcc and hcp hollow sites with a H:Fe ratio of 1:2, as seen in the simplified ball model with a single atomic Fe layer in Supplementary Fig. 3b. An alternative is the adsorption of H in 3 out of 4 hollow sites of the same type, see Supplementary Fig. 3c, resulting in a H concentration of 0.75 with respect to one Fe atom in the unit cell. Since hydrogen vacancies (bright protrusions) on top of the H1-Fe exhibit the same geometrical shape, indicated by white triangles pointing along the $[11\bar{2}]$ direction in Supplementary Fig. 3a, we are able to infer the positions from which the H atoms are possibly missing in the structural models, marked by solid red circles in Supplementary Figs. 3b and c. The 0.98 nm period H2-Fe supercell comprises 13 Fe atoms, and it is rotated with respect to the high-symmetry lines of the Ir (111) surface by about ±14°, forming two rotational domains as can be seen, e.g., for the two islands in Fig. 2 of the main text. Several different H-loaded states can be constructed for this supercell, two of which are shown in Supplementary Figs. 3d and 3e with 16 and 9 H atoms per unit cell in different adsorption sites in this single-layer model.

Supplementary Note 2 | Identification of skyrmions and defects.

To distinguish structural and magnetic features in STM, it is useful to compare topographic constant-current images and simultaneously acquired differential tunneling conductance (dI/dU) maps. For the measurement shown in Fig. 3 of the main text, this data is displayed in Supplementary Fig. 4. In the topography image the defects due to incomplete formation of the H1-Fe phase are imaged as bright protrusions, whereas in the dI/dU maps they appear as darker dots. In contrast, in this measurement with a magnetic tip being dominantly sensitive to the in-plane sample magnetization component, the magnetic contribution to the signals appears similar in both measurement channels.

Supplementary Note 3 | VASP calculations.

For the pristine Fe-DL, different stacking orders of the layers were compared as shown in Supplementary Table 1. Ideally the fcc Ir lattice grows in A/B/C stacking along the (111) direction, with an interlayer distance of $\sqrt{2/3}a = 2.21$ Å. The distance between the top Ir layer and the one below it was slightly larger than this value (2.28 Å) and only very weakly depended on the considered H adsorption sites and concentrations. On the other hand, the Ir-Fe1 and Fe1-Fe2 distances were reduced by approximately 10 pm and 40 pm compared to the ideal interlayer distance due to the smaller bulk lattice constant of Fe, which is also responsible for the appearance of reconstruction lines in the experiments. We only took into account stackings preserving the $C_{3\mathrm{v}}$ symmetry of the system, since the hydrogenated structures also displayed this symmetry due to the disappearance of reconstruction lines. Reconstructed geometries in the same system have been investigated recently in ref. 5. We found that the stacking continuing the A/B/C order has the lowest energy, and the non-

collinear magnetic structure was only investigated for this stacking with the SKKR code, where the obtained spin spiral period was found to be in reasonable agreement with the experiments. Therefore, in the following calculations only this stacking was considered, although we cannot exclude that the H adsorption also changes the stacking of the Fe layers.

For a full monolayer of H coverage, we considered nine possible adsorption sites as summarized in Supplementary Table 2, all of them preserving the $C_{3\mathrm{v}}$ symmetry, which was also observed in the experiments. The lowest energy corresponds to the fcc adsorption site on the surface (Fe2-Vac A). In this case, the top Fe2 layer moves slightly away from the substrate (by about 8 pm), similarly to the H adsorption effect on Fe/Ir(001) investigated in ref. 6. However, the SKKR calculations demonstrated that this adsorption site actually leads to a decrease in the magnetic period, contrary to the experimental findings. Furthermore, the experimental observations discussed in Supplementary Note 1 indicated that the H1-Fe and H2-Fe structures may be due to the adsorption of H at different distances from the surface. Therefore, we also determined the magnetic interactions between the Fe atoms for H in the octahedral position between the Fe layers (Fe1-Fe2 A) and at the Ir-Fe interface (Ir-Fe1 C). Although these states have significantly higher energies, in earlier calculations performed for the surface of bulk bcc Fe it was found[7] that the H atoms may overcome energy differences of similar magnitude when diffusing into the metal. The distance between the Fe layers in these configurations was increased by about 30 and 20 pm with respect to the pristine Fe-DL, leading to a significant enhancement of the magnetic period. Furthermore, for H between the substrate and the magnetic layers the Ir-Fe distance also increased by about 20 pm, which was found to be responsible for the weakening of the Dzyaloshinsky–Moriya interaction and the formation of the ferromagnetic ground state as discussed in the main text.

Supplementary Table 2 also displays the Bader charges[8,9,10] for H, indicating that in most considered adsorption sites charge is transferred from the Fe to the H atoms[7,11]. This charge transfer is maximal for H between the magnetic layers with 4 or 6 Fe neighbours and lower for H at the Ir-Fe and Fe-Vac interfaces with only 3 Fe neighbours. Finally, the obtained Bader charge of H is below 1 if it has only a single Fe neighbour (Fe2-Vac C, Ir-Fe1 B), which can be explained by the H orbitals extending into the vacuum or an opposite direction of charge transfer between H and Ir. The charge transfer is accompanied by a decrease of the Fe magnetic moments, especially in the layers directly neighbouring the H atom.

Finally, we also investigated the effect of different H coverages to obtain a more accurate description of the H1-Fe structure, which was found to exhibit a $\mathrm{p}(2 \times 2)$ atomic superstructure in the experiments. The results are summarized in Supplementary Table 3, with the parameters averaged over atoms nominally belonging to the same atomic layer. We assumed that the H is adsorbed into the octahedral positions between the two Fe layers (Fe1-Fe2 A), since earlier it was found that this leads to a significant increase of the magnetic period. We considered 1, 2 or 3 H atoms in these sites in the $\mathrm{p}(2 \times 2)$ unit cell. As expected, both the increase in the Fe1-Fe2 interlayer distance and the decrease in the magnetic moments is monotonic in the H coverage, which also leads to a monotonic increase in the spin spiral period as shown in Fig. 4b in the main text.

Supplementary Note 4 | SKKR self-consistent calculations.

For comparison with the VASP calculations, the spin magnetic moments determined in the ferromagnetic configuration and the charges on the H atoms from the SKKR calculations are summarized in Supplementary Table 4. As mentioned in the Methods section, the Wigner–Seitz radii of the atomic spheres were determined in such a way as to minimize the overlap between the spheres. In the case of the H atoms, this meant that the charge inside the sphere must approximate the Bader charge determined from VASP calculations, otherwise it would be possible that the atomic sphere cuts deeply inside the electron cloud of the neighbouring Fe atoms. With such a choice of the Wigner–Seitz radii, it is clear that the SKKR calculations reproduce the decrease in the magnetic moments due to H adsorption observed in the VASP calculations. A slight deviation from this trend can be observed between 0.00 ML and 0.25 ML concentrations for the magnetic moments, but this can be explained by the fact that no H atomic sphere was included in the calculations for the Fe-DL at 0.00 ML coverage.

Supplementary Note 5 | Dependence of the spin spiral period on *ab initio* calculation parameters.

During the *ab initio* calculations it is possible to tune all calculation parameters independently of each other, enabling one to separate the contribution of different effects on the spin spiral period. As shown in Supplementary Fig. 5, we performed investigations for the model describing the H1-Fe system (0.50 ML H coverage in the Fe1-Fe2 A position) by changing the distance between the Fe layers and the H concentration. Supplementary Figure 5a displays that modifying the interlayer distance by about 15 pm enhances the magnetic period by about a factor of four. A similar correlation between the substrate-magnetic layer distance and the magnetic period has been pointed out in several earlier publications[1,12,13,14,15,16], generally in connection with the decreased hybridization as mentioned in the main manuscript. It is important to note that there is some uncertainty in the interlayer distances determined from *ab initio* calculations due to the different types of approximations involved. For example, we observed that using the Perdew-Burke-Ernzerhof[17] (PBE) parametrization of the exchange-correlation potential instead of the PW91[18] method mentioned in the Methods section changes the Fe1-Fe2 distance by about 2 pm in the VASP calculations. Switching to the local density approximation (LDA) from the generalized gradient approximation (GGA) is expected to have an even larger influence[12]. For a fixed interlayer distance, the magnetic period determined from the SKKR method also depends on the type of exchange-correlation potential. However, the predictions that the inclusion of H between the Fe layers or at the Ir-Fe interface increases the interlayer distance, and the enhancement of this distance leads to a larger magnetic period, are independent of the exact forms of approximations involved in the calculations; therefore, they may be used for a comparison with the experimental observations.

Tuning the H concentration in a fixed geometry may have various effects on the calculated magnetic period depending on the adsorption sites, as shown in Supplementary Figs. 5b,c. For the H between the Fe atomic layers (see Supplementary Fig. 5b), an increase of the magnetic period by about a factor of two is obtained between 0.00 ML and 1.00 ML of H. This indicates that in this geometry the hybridization between Fe and H atomic orbitals has an analogous effect to the increase of the Fe interlayer distance. In contrast, Supplementary Fig. 5c shows the concentration dependence if H is adsorbed on top of the Fe layers in the fcc (Fe2-Vac A) positions. At 0.00 ML concentration the spin spiral period is somewhat larger than for the Fe-DL due to the slightly increased Fe1-Fe2 distance – see Supplementary Table 2 –, around 1.9 nm compared to 1.4 nm. On the other hand, the H-Fe

hybridization counteracts this effect in this case and leads to a reduction in the spin spiral period as the H concentration is increased, also displayed in Fig. 4a of the main text, with a 1.3 nm period for 1.00 ML coverage in this adsorption position.

Supplementary Note 6 | Connection between *ab initio* interaction parameters, the simplified model and the micromagnetic description.

As mentioned in the Methods section, the interaction coefficients between the spins were determined from the relativistic torque method[19] for all neighbours within a radius of $8a$. The classical spin Hamiltonian describing the system reads

$$H = -\frac{1}{2}\sum_{i \neq j} \mathbf{S}_i \mathbf{J}_{ij} \mathbf{S}_j - \sum_i \mathbf{S}_i \mathbf{K}_i \mathbf{S}_i - \sum_i \mu_{\mathrm{s},i} \mathbf{S}_i \mathbf{B}, \tag{1}$$

with $\mathbf{S}_i$ unit vectors representing the spins at site i, $\mu_{\mathrm{s},i}$ layer-dependent spin magnetic moments and $\mathbf{B}$ the external magnetic field. The $\mathbf{K}_i$ layer-dependent on-site anisotropy tensor may be characterized by a single value K_i^{zz} in the considered symmetry class. From the $\mathbf{J}_{ij}$ interaction tensor it is possible to determine the Heisenberg exchange interactions $J_{ij} = \frac{1}{3} J_{ij}^{\alpha\alpha}$ and the Dzyaloshinsky–Moriya vectors $D_{ij}^{\alpha} = \frac{1}{2} \varepsilon^{\alpha\beta\gamma} J_{ij}^{\beta\gamma}$; summation over Cartesian indices α, β, γ appearing twice is understood in the expressions. For the Fe-DL, H1-Fe and H2-Fe structures discussed in the main text, these parameters are displayed in Supplementary Fig. 6. Both the Heisenberg and the Dzyaloshinsky–Moriya exchange coefficients display an oscillatory decay, typical for RKKY-like interactions[20,21,22] in ultrathin magnetic systems on heavy metal substrates[23]. The difference between the diagonal components of the interaction tensor also contributes to the total magnetic anisotropy energy as $E_{\mathrm{ani},i} = \frac{1}{2}\sum_j \left(J_{ij}^{zz} - J_{ij}^{xx}\right) + K_i^{zz}$.

Considering interaction parameters with many different neighbours is essential in correctly describing the high-energy behaviour of the system[14], for example, in simulations performed at higher temperatures[24]. It was demonstrated recently that if the interaction parameters determine different magnetic periods in the different atomic layers, this can lead to a significant modulation of the spin spiral wavelength as the temperature is increased[25]. However, the investigations for the present system were carried out at low temperature and the shape of the equilibrium spin structures was considered, and it is expected that the simplified model given in Eq. (1) and Fig. 5 of the main text can correctly account for these effects. As demonstrated in Supplementary Fig. 7, we indeed obtained a very similar zero-temperature phase diagram for the H1-Fe system to Fig. 5 if the calculations were carried out with the *ab initio* interaction parameters instead of the simplified model, similarly to the observation in ref. 26.

The simplified model parameters in Eq. (1) were determined as follows. The energy of the spin spirals was calculated for harmonic configurations in the plane defined by the surface normal vector $\mathbf{n}$ and an in-plane unit vector $\mathbf{e}$,

$$\mathbf{S}_i = \mathbf{n} \cos \mathbf{k}\mathbf{R}_i + \mathbf{e} \sin \mathbf{k}\mathbf{R}_i, \tag{2}$$

which transforms into Eq. (2) in the Methods section for cycloidal spin spirals where $\mathbf{e}$ is along the wave vector direction. For a fixed direction of $\mathbf{e}$ replacing $\mathbf{k}$ by $-\mathbf{k}$ switches the chirality of the spiral. The energy per spin reads[27]

$$\frac{1}{N}E_{\mathrm{SS}}(\mathbf{k}) = \frac{1}{n_{\mathrm{layer}}}\sum_{\mathrm{layers}}\left(E_{\mathrm{symm},i} + E_{\mathrm{antisymm},i} + E_{\mathrm{ani},i}\right), \tag{3}$$

$$E_{\mathrm{symm},i} = -\frac{1}{2}\sum_{\mathbf{R}_j-\mathbf{R}_i}\frac{1}{2}(\mathbf{eJ}_{ij}\mathbf{e} + \mathbf{nJ}_{ij}\mathbf{n})\cos\left[\mathbf{k}(\mathbf{R}_j - \mathbf{R}_i)\right], \tag{4}$$

$$E_{\mathrm{antisymm},i} = -\frac{1}{2}\sum_{\mathbf{R}_j-\mathbf{R}_i}\mathbf{D}_{ij}(\mathbf{e}\times\mathbf{n})\sin\left[\mathbf{k}(\mathbf{R}_j - \mathbf{R}_i)\right], \tag{5}$$

$$E_{\mathrm{ani},i} = -\frac{1}{2}(\mathbf{eK}_i\mathbf{e} + \mathbf{nK}_i\mathbf{n}), \tag{6}$$

which has to be compared to that of the ferromagnetic state,

$$\frac{1}{N}E_{\mathrm{FM}} = \frac{1}{n_{\mathrm{layer}}}\sum_{\mathrm{layers}}\left(-\frac{1}{2}\sum_{\mathbf{R}_j-\mathbf{R}_i}\mathbf{nJ}_{ij}\mathbf{n} - \mathbf{nK}_i\mathbf{n}\right). \tag{7}$$

The dispersion relations were calculated for 128×128 $\mathbf{k}$ points in the Brillouin zone for the Fe-DL and 512×512 $\mathbf{k}$ points for the hydrogenated structures. The energy difference between the spin spiral at zero wave vector and the energy of the ferromagnetic state is half of $E_{\mathrm{ani},i}$ averaged over the layers, which was identified with half of the anisotropy parameter K in Eq. (1).

If the spin spiral dispersion relations are calculated from the model given by Eq. (1), the symmetric and antisymmetric contributions read

$$E_{\mathrm{symm}} = -J_1\left[\cos(k^x a) + 2\cos\left(\frac{1}{2}k^x a\right)\cos\left(\frac{\sqrt{3}}{2}k^y a\right) - 3\right]$$
$$-J_2\left[\cos\left(\sqrt{3}k^y a\right) + 2\cos\left(\frac{3}{2}k^x a\right)\cos\left(\frac{\sqrt{3}}{2}k^y a\right) - 3\right], \tag{8}$$

$$E_{\mathrm{antisymm},xz} = -D\left[\sin(k^x a) + \sin\left(\frac{1}{2}k^x a\right)\cos\left(\frac{\sqrt{3}}{2}k^y a\right)\right], \tag{9}$$

$$E_{\mathrm{antisymm},yz} = -\sqrt{3}D\cos\left(\frac{1}{2}k^x a\right)\sin\left(\frac{\sqrt{3}}{2}k^y a\right), \tag{10}$$

for spin spirals rotating in the xz and yz planes, respectively; z denotes the out-of-plane direction, x is along the nearest-neighbour $(1\bar{1}0)$ and y is along the next-nearest-neighbour $(11\bar{2})$ direction. Note that the symmetric energy contribution was shifted to be equal to zero at zero wave vector. The interaction parameters collected in Table 1 in the main text were determined by fitting the two-dimensional dispersion relations with Supplementary Eqs. (8)-(10) in a range around the center of the Brillouin zone including the spin spiral energy minima. The model does not approximate the spin spiral dispersion relation well at higher wave vectors; therefore, it is not expected to correctly account for the high-temperature behaviour as discussed above.

The simplified atomic model essentially corresponds to a micromagnetic description formulated on a hexagonal lattice, which is also expected to be only applicable at low wave vectors and (unless the temperature dependence of the parameters is considered) at zero temperature. The micromagnetic model is defined by the free-energy density[28,29]

$$f_0 = \mathcal{J}_1(\nabla \mathbf{S})^2 + \mathcal{J}_2(\nabla^2 \mathbf{S})^2 + \mathcal{D} w_D(\mathbf{S}) - \mathcal{K}(S^z)^2 - M\mathbf{BS}, \tag{11}$$

with $\mathbf{S}$ the unit length vector field, M the magnetization, $\mathcal{J}_1$ the exchange stiffness, $\mathcal{J}_2$ the higher-order exchange parameter, $\mathcal{D}$ the Dzyaloshinsky–Moriya interaction and $\mathcal{K}$ the anisotropy constant. The energy density of the Dzyaloshinsky–Moriya interaction reads

$$w_D(\boldsymbol{S}) = S^z \partial_x S^x - S^x \partial_x S^z + S^z \partial_y S^y - S^y \partial_y S^z, \tag{12}$$

with the sign convention that $\mathcal{D} > 0$ prefers left-handed rotation.

The connection between the simplified model parameters and the micromagnetic parameters may be obtained by expanding Supplementary Eqs. (8)-(10) at low wave vectors, yielding

$$E_{\text{symm}} = \frac{3}{4} a^2 (J_1 + 3J_2) \mathbf{k}^2 - \frac{9}{192} a^4 (J_1 + 9J_2)(\mathbf{k}^2)^2 + \mathcal{O}(k^6), \tag{13}$$

$$E_{\text{antisymm},xz} = -\frac{3}{2} aDk^x + \mathcal{O}(k^3), \tag{14}$$

$$E_{\text{antisymm},yz} = -\frac{3}{2} aDk^y + \mathcal{O}(k^3). \tag{15}$$

Comparing Supplementary Eq. (11) after spatial Fourier transformation to Supplementary Eqs. (13)-(15) leads to the correspondence

$$\mathcal{J}_1 = \frac{1}{V_{\text{WS}}} \frac{3}{4} a^2 (J_1 + 3J_2), \tag{16}$$

$$\mathcal{J}_2 = -\frac{1}{V_{\text{WS}}} \frac{9}{192} a^4 (J_1 + 9J_2), \tag{17}$$

$$\mathcal{D} = \frac{1}{V_{\text{WS}}} \frac{3}{2} aD, \tag{18}$$

$$\mathcal{K} = \frac{1}{V_{\text{WS}}} K, \tag{19}$$

$$M = \frac{1}{V_{\text{WS}}} \mu_{\text{s}}, \tag{20}$$

where V_{WS} denotes the volume occupied by a single Fe atom. This was determined from the Wigner–Seitz radii used for the SKKR calculations by averaging over the two magnetic layers, and can be well approximated by $V_{\text{WS}} \approx \frac{\sqrt{3}}{2} a^2 t$, where the layer thickness t is the distance between the outer Fe2 and the top Ir layer divided by two.

The micromagnetic interaction coefficients are summarized in Supplementary Table 5 for the systems displayed in Table 1 of the main text. Supplementary Equation (16) explains the importance of the factor $f = (J_1 + 3J_2)/J_1$; for negative values one has $\mathcal{J}_1 < 0$, meaning that the micromagnetic model is only stabilized by the higher-order $\mathcal{J}_2$ term, and the Heisenberg exchange interactions already determine an energy minimum at finite wave vector $k = \sqrt{-\frac{\mathcal{J}_1}{2\mathcal{J}_2}}$.

Supplementary References

1. Heinze, S. et al. Spontaneous atomic-scale magnetic skyrmion lattice in two dimensions. *Nat. Phys.* **7**, 713-718 (2011).
2. Hsu, P.-J. et al. Guiding Spin Spirals by Local Uniaxial Strain Relief. *Phys. Rev. Lett.* **116**, 017201 (2016).
3. Reimer, P. M., Zabel, H., Flynn, C. P. & Dura, J. A. Extraordinary alignment of Nb films with sapphire and the effects of added hydrogen. *Phys. Rev.* B **45**, 11426-11429 (1992).
4. Arman, M. A. et al. Adsorption of hydrogen on stable and metastable Ir(100) surfaces. *Surf. Sci.* **656**, 66-76 (2017).
5. Hauptmann, N. et al. Revealing the correlation between real-space structure and chiral magnetic order at the atomic scale. Preprint at arXiv:1712.02717 (2017).
6. Máca, F., Kudrnovský, J., Drchal, V. & Redinger, J. Influence of oxygen and hydrogen adsorption on the magnetic structure of an ultrathin iron film on an Ir(001) surface. *Phys. Rev.* B **88**, 045423 (2013).
7. Jiang, D. E. & Carter, E. A. Diffusion of interstitial hydrogen into and through bcc Fe from first principles. *Phys. Rev.* B **70**, 064102 (2004).
8. Henkelman, G., Arnaldsson, A. & Jónsson, H. A fast and robust algorithm for Bader decomposition of charge density. *Comput. Mater. Sci.* **36**, 354-360 (2006).
9. Sanville, E., Kenny, S. D., Smith, R. & Henkelman, G. An improved grid-based algorithm for Bader charge allocation. *J. Comp. Chem.* **28**, 899-908 (2007).
10. Tang, W., Sanville, E. & Henkelman, G. A grid-based Bader analysis algorithm without lattice bias. *J. Phys.: Condens. Matter* **21**, 084204 (2009).
11. Bessarab, P. F., Uzdin, V. M. & Jónsson, H. Effect of hydrogen adsorption on the magnetic properties of a surface nanocluster of iron. *Phys. Rev.* B **88**, 214407 (2013).
12. Kudrnovský, J., Máca, F., Turek, I. & Redinger, J. Substrate-induced antiferromagnetism of a Fe monolayer on the Ir(001) surface. *Phys. Rev.* B **80**, 064405 (2009).
13. von Bergmann, K. et al. Observation of a Complex Nanoscale Magnetic Structure in a Hexagonal Fe Monolayer. *Phys. Rev. Lett.* **96**, 167203 (2006).
14. Dupé, B., Hoffmann, M., Paillard, C. & Heinze, S. Tailoring magnetic skyrmions in ultra-thin transition metal films. *Nat. Commun.* **5**, 4030 (2014).
15. Simon, E., Palotás, K., Rózsa, L., Udvardi, L. & Szunyogh, L. Formation of magnetic skyrmions with tunable properties in PdFe bilayer deposited on Ir(111). *Phys. Rev.* B **90**, 094410 (2014).
16. Rózsa, L., Udvardi, L., Szunyogh, L. & Szabó, I. A. Magnetic phase diagram of an Fe monolayer on W(110) and Ta(110) surfaces based on *ab initio* calculations. *Phys. Rev.* B **91**, 144424 (2015).
17. Perdew, J. P., Burke, K. & Ernzerhof, M. Generalized Gradient Approximation Made Simple. *Phys. Rev. Lett.* **77**, 3865-3868 (1997).
18. Perdew, J. P. & Wang, Y. Accurate and simple analytic representation of the electron-gas correlation energy. *Phys. Rev.* B **45**, 13244-13249 (1992).
19. Udvardi, L., Szunyogh, L., Palotás, K. & Weinberger, P. First-principles relativistic study of spin waves in thin magnetic films. *Phys. Rev.* B **68**, 104436 (2003).
20. Ruderman, M. A. & Kittel, C. Indirect Exchange Coupling of Nuclear Magnetic Moments by Conduction Electrons. *Phys. Rev.* **96**, 99-102 (1954).
21. Kasuya, T. A Theory of Metallic Ferro- and Antiferromagnetism on Zener's Model. *Prog. Theor. Phys.* **16**, 45-57 (1956).
22. Yosida, K. Magnetic Properties of Cu-Mn Alloys. *Phys. Rev.* **106**, 893-898 (1957).

23. Khajetoorians, A. A. et al. Tailoring the chiral magnetic interaction between two individual atoms. *Nat. Commun.* **7**, 10620 (2016).
24. Rózsa, L., Simon, E., Palotás, K., Udvardi, L. & Szunyogh, L. Complex magnetic phase diagram and skyrmion lifetime in an ultrathin film from atomistic simulations. *Phys. Rev.* B **93**, 024417 (2016).
25. Finco, A. et al. Temperature-Induced Increase of Spin Spiral Periods. *Phys. Rev. Lett.* **119**, 037202 (2017).
26. von Malottki, S., Dupé, B., Bessarab, P. F., Delin, A. & Heinze, S. Enhanced skyrmion stability due to exchange frustration. *Sci. Rep.* **7**, 12299 (2017).
27. Rózsa, L. et al. Skyrmions with Attractive Interactions in an Ultrathin Magnetic Film. *Phys. Rev. Lett.* **117**, 157205 (2016).
28. Michelson, A. Phase diagrams near the Lifshitz point. I. Uniaxial magnetization. *Phys. Rev.* B **16**, 577-584 (1977).
29. Rózsa, L. et al. Formation and stability of metastable skyrmionic spin structures with various topologies in an ultrathin film. *Phys. Rev.* B **95**, 094423 (2017).
